# MicroRNAs as the critical regulators of Forkhead box protein family during gynecological and breast tumor progression and metastasis

**DOI:** 10.1186/s40001-023-01329-7

**Published:** 2023-09-09

**Authors:** Negin Taghehchian, Malihe Lotfi, Amir Sadra Zangouei, Iman Akhlaghipour, Meysam Moghbeli

**Affiliations:** 1 Medical Genetics Research Center, Mashhad University of Medical Sciences, Mashhad, Iran; 2 Student Research Committee, Faculty of Medicine, Mashhad University of Medical Sciences, Mashhad, Iran; 3 Department of Medical Genetics and Molecular Medicine, School of Medicine, Mashhad University of Medical Sciences, Mashhad, Iran

**Keywords:** Gynecological cancer, Breast cancer, miRNAs, FOX, Diagnosis

## Abstract

Gynecological and breast tumors are one of the main causes of cancer-related mortalities among women. Despite recent advances in diagnostic and therapeutic methods, tumor relapse is observed in a high percentage of these patients due to the treatment failure. Late diagnosis in advanced tumor stages is one of the main reasons for the treatment failure and recurrence in these tumors. Therefore, it is necessary to assess the molecular mechanisms involved in progression of these tumors to introduce the efficient early diagnostic markers. Fokhead Box (FOX) is a family of transcription factors with a key role in regulation of a wide variety of cellular mechanisms. Deregulation of FOX proteins has been observed in different cancers. MicroRNAs (miRNAs) as a group of non-coding RNAs have important roles in post-transcriptional regulation of the genes involved in cellular mechanisms. They are also the non-invasive diagnostic markers due to their high stability in body fluids. Considering the importance of FOX proteins in the progression of breast and gynecological tumors, we investigated the role of miRNAs in regulation of the FOX proteins in these tumors. MicroRNAs were mainly involved in progression of these tumors through FOXM, FOXP, and FOXO. The present review paves the way to suggest a non-invasive diagnostic panel marker based on the miRNAs/FOX axis in breast and gynecological cancers.

## Background

Breast cancer (BC) is the most frequently diagnosed (2.3 million cases or 11.7% of all cancers) and the fifth cancer-related mortality (685,000 death or 6.9% of all cancer) worldwide [1]. Invasive ductal carcinoma is the main histological type of BC with prevalence rate of 50–80% [2–4]. Gynecological tumors are one of the most frequent and the major causes of cancer-related death in women worldwide. The five main types of gynecological cancer are cervical, uterine, vaginal, vulvar, and ovarian. Gynecological tumors incidence is about 1.4 of 19 million of all new cases with 0.7 out of 10 million deaths per year. Cervical cancer (CC) is the fourth cancer-related mortality in women globally. It is estimated that there were over 600,000 (3.1%) new cervical cancer cases and 342,000 (3.4%) deaths in 2020. Vaginal cancer is a rare cancer that accounts for 0.1% of new cases deaths globally. Vulvar cancer is also a rare gynecological cancer with an estimated incidence and mortality rate of 0.2% in 2020. Ovarian cancer also accounts for 1.6% of all new cases and 2.1% death worldwide [1].

Chemotherapy is the first therapeutic option for many types of cancers. However, chemo resistance remains still a challenge in tumor therapy [5, 6]. Over 90% of cancer-related deaths occur in patients with drug resistance [7, 8]. More than half of all patients suffering from cancer will undergo chemo-treatment. Resistance to chemotherapy develops in 50–96% of cancer patients within 6–9 months of treatment [9, 10]. This is a major obstacle to achieving a high rate of complete pathological response during cancer treatment [11]. About 85–90% of chemotherapy failures have been reported in breast and ovarian tumors [12]. The 5-year survival rate of BC is about 80–90%, and early diagnosis can give the best treatment results [13]. About 80–90% of endometrial cancers are early stage with good prognosis [14]. Around 20% of endometrial cancer patients who are treated with chemotherapy alone, experience regional recurrence [15]. Early diagnosis improves the survival rates of ovarian cancer patients up to 70% [16]. Absence of specific symptoms in the early stages of ovarian cancer and lack of effective biomarker screening are major reasons for the increasing number of patients being diagnosed with advanced stages and poor prognosis [17]. The 7-year survival rate for patients with end-stage ovarian cancer treated with chemotherapy is only 9% [18]. Tumor relapse due to treatment failure occurs in about 70% of the ovarian cancer cases [19]. About 30–50% of cervical cancer patients develop treatment failure due to regional recurrence [20]. Therefore, early detection of gynecological and breast tumors can be an important way to find the most efficient treatment. To find novel and efficient early detection markers, it is necessary to assess the molecular mechanisms involved in these cancers.

Forkhead box (FOX) transcription factors are involved in regulation of a wide variety of cellular mechanisms, such as cell proliferation, metabolism, migration, and tumor progression [21]. Mammalian FOX proteins are categorized into 19 subgroups including (FOXA to FOXS) based on sequence similarity outside and inside of the forkhead box [22]. Fox proteins possess highly conserved DNA-binding domains (FOX–DBD) but have different properties and functions [23]. Forkhead domain (FHD) structurally contains three β-strands, three n-ter α-helices (H1–3), and two loops, constructing butterfly winged helix in its C-terminal region (W1–2) [24]. FHD could interact with specific sequences, including the major groove of DNA and the H3 helix (recognition helix) [25]. In addition, the specificity of Forkheads DNA-binding is related to the variable region located on the junction of helices H2 and H3 and wings W1 and W2, which links to bases in the minor groove of DNA [26]. Association between FOXA1 and its target sequences has indicated that wings could regulate the DNA-binding affinity and specificity of the nominated domain [27]. This domain is also accountable for nuclear transportation. FOXE1, FOXA2, FOXF2, and FOXP3 have two nuclear localization sequences (NLS) at both ends of the domain site, which were located in H1 and W2 [28, 29]. FOX deregulation can be associated with diabetes mellitus, congenital disorders, and cancers. There was FOXC1 up-regulation in cervical cancer that was correlated with OS, stage, and metastasis. Down-regulation of FOXC1 inhibited cell proliferation and invasion through modulating the AKT cascade [30]. FOXA1 could inhibit the EMT process and angiogenesis by VEGF inhibition in cervical cancer [31]. FOXA2 plays a tumor-suppressive role in endometrial carcinoma which could suppress cell cycle progression through Myc [32]. FOXC2 regulates the MAPK and Akt pathways to down-regulate Bcl-2 while up-regulate Bax and CASP3 that intervene in the CDDP resistance of ovarian cancer [33]. Suppression of FoxM1 is a critical strategy to overcome the metastatic breast cancer progression [34]. MicroRNAs (miRNAs) are non-coding RNAs involved in prost-transcriptional regulation by mRNA degradation or translational inhibition [35]. They are also the key regulators of cell cycle, apoptosis, and differentiation [36]. MiRNAs biogenesis begins in the nucleus with the generation of polyadenylated and capped primary miRNAs (pri-miRNAs) transcripts via RNA polymerase II (PolII) [37]. Pri-miRNAs are further processed via Drosha/DGCR8 complex into single hairpin precursor miRNAs (pre-miRNAs) [38]. Pre-miRNAs are exported into the cytoplasm via the exportin 5 (XPO5) and cleaved by Dicer. This process contains the cleavage of the terminal loop, which leads to forming of a mature miRNA duplex intermediate [39, 40]. Mature miRNA duplex consists of two strands that can be loaded into the Argonaute (AGO) proteins. Moreover, the guide strand is located in the RNA-induced silencing complex (RISC), where it could target the complementary 3′-untranslated regions (UTR) of target mRNAs [41]. MiRNAs deregulations have been reported to be associated with tumor progression and drug resistance [42–44]. MiRNAs have a good potential as diagnostic biomarkers for the early detection of cancers [45, 46]. MiRNA profiles can distinguish not only the tissue of origin, but also the various subtypes of a particular cancer [45]. They have also a high stability in serum and blood plasma [47, 48]. Therefore, miRNAs can be used as minimally invasive tumor biomarkers [49, 50]. It has been shown that miRNAs are important regulators of the FOX proteins in cervical and breast tumors [51, 52]. Considering the pivotal role of FOX proteins in gynecological and breast tumors, we discussed the role of miRNAs–FOX axis as an important molecular mechanism involved in progression and metastasis in these tumors (Table 1).

**Table 1:** Role of miRNAs in regulation of gynecological and breast cancers via FOX targeting

Study	Tumor type	Gene	Target	Samples	Function
Lin [52]	Breast	miR-96	FOXO3a	23 tumor samples 23 normal samples MCF-7, ZR-75-30, BT549, Bcap37, MDA-MB435, SKBR3, MDA-MB453 and T47D cell lines	Oncogene
Shen [55]	Breast	miR-204	FOXA1	MCF-7 cell line	Tumor suppressor
Salem [56]	Ovarian	miR-590-3p	FOXA2	58 tumor samples 13 plasma samples ES-2, SKOV3.ip1,OVCAR3, and HEY cell lines	Oncogene
Xu [63]	Endometrial	miR-495	FOXC1	10 tumor samples 5 normal samples AN3CA and KLE cell lines	Tumor suppressor
Zhang [72]	Cervical	miR-30a-5p	FOXD1	50 tumor samples 50 normal samples HeLa, HT-3, C33A and SiHa cell lines	Tumor suppressor
Yang [75]	Cervical	miR‑128‑3p	FOXD3	60 tumor samples 60 normal samples C33A, HeLa, HT-3 and SiHa cell lines	Tumor suppressor
Zhang [76]	Breast	miR-182	FOXF2	55 tumor samples 55 normal samples MCF-7 and MDA-MB-231 cell lines	Oncogene
Wang [77]	Ovarian	miR-182-5p	FOXF2	47 tumor samples 47 normal samples SKOV3, HO8910, A2780, OVCAR, and HOSEpiC cell lines	Oncogene
Zeng [86]	Cervical	miR-200b	FoxG1	30 tumor samples 30 normal samples HeLa and C33A cell lines	Oncogene
Gao [88]	Breast	miR-365-3p	FOXK1	93 tumor samples 93 normal samples MCF-7 and MDA-MB-231 cell lines	Oncogene
Zhang [101]	Breast	miR-26b	FOXM1	MDA-MB-231, MDA-MB-436, MDA-MB-468, MDA-MB-157, MDA-MB-435 and BT549 cell lines	Oncogene
Bayraktar [105]	Breast	miR-34a	FOXM1	MDA-MB-231, MDA-MB-436, MDA-MB-468, BT-483, SUM-149, and HCC1937 cell lines	Tumor suppressor
Yuan [107]	Breast	miR-802	FOXM1	20 tumor samples 20 normal samples MCF-7, MDA-MB-453, MDA-MB-468 and ZR-75–1 cell lines	Tumor suppressor
Li [108]	Cervical	miR-342-3p	FOXM1	27 tumor samples 27 normal samples HeLa, Caski, and C33A cell lines	Tumor suppressor
Liang [109]	Cervical	miR-4429	FOXM1	102 tumor samples 102 normal samples CaSki, ME-180, C33A, and SiHa cell lines	Tumor suppressor
Li [110]	Ovarian	miR-149-5p	FOXM1	SKOV3 and A2780 cell lines	Tumor suppressor
Hong [111]	Cervical	miR-320a	FOXM1	48 tumor samples 48 normal samples SiHa, HeLa, CaSki, C-33A, and MS751cell lines	Tumor suppressor
Gao [112]	Cervical	miR-361-5p	FOXM1	66 tumor samples 66 normal samples SiHa, HeLa, C33a, Me180 and Ms751 cell lines	Tumor suppressor
Dong [113]	Breast	miR-876-5p	FOXM1	MCF-7, BT-549, MDA-MB-231, and SKBR3 cell lines	Tumor suppressor
Tan [114]	Breast	miR-671-5p	FOXM1	30 tumor samples 30 normal samples MDA-MB-231, Hs578T, SKBR3, BT-20, MDA-MB-468, MCF-7, and T47D cell lines	Tumor suppressor
He [115]	Cervical	miR-216b	FOXM1	150 tumor samples 150 normal samples HCC94, HeLa (Cat, SiHa, Ca Ski, and C33A cell lines	Tumor suppressor
Hu [116]	Cervical	miR‑197	FOXM1	46 tumor samples 46 normal samples HeLa, C33A, CaSki and SiHa cell lines	Tumor suppressor
Shi [117]	Cervical	miR‑320	FOXM1	36 tumor samples 36 normal samples HeLa, CaSki, C33A and SiHa cell lines	Tumor suppressor
Xia [118]	Cervical	miR-374b	FOXM1	48 tumor samples 48 normal samples SiHa, Hela and CaSki cell lines	Tumor suppressor
Dai [119]	Cervical	miR-203a-5p	FOXN2	47 tumor samples 47 normal samples SiHa, C-4-I, Ca-Ski and C-33-A cell lines	Oncogene
Xu [123]	Cervical	miR-181a	FOXO1	C33A, HeLa229, MS751, HCC94, HeLa, HT-3, SiHa, CaSKi, and ME-180 cell lines	Oncogene
Xu [124]	Cervical	miR-135b	FOXO1	C33A, HCC94, HeLa, HT-3, SiHa and CaSKi cell lines	Oncogene
Yang [125]	Cervical	miR-96	FOXO1	83 tumor samples 11 normal samples C41, C33A, HeLa, CaSki, MS751, SiHa and HT-3 cell lines	Oncogene
Hou [51]	Cervical	miR-196a	FOXO1	102 tumor samples 10 normal samples MS751, C33A, HeLa, HeLa229, SiHa, HCC94, CaSKi, HT-3 and ME-180 cell lines	Oncogene
Li [128]	Breast	miR-29c	FOXO1	79 tumor samples 16 normal samples 20 healthy serum 79 BC serum MCF-7, MDA-MB-231, and MDA-MB-436 cell lines	Tumor suppressor
Liu [129]	Breast	miR-9	FOXO1	83 tumor samples 83 normal samples BT-549, MDA-MB-231, MCF7, BT-474, MDA-MB-453, MDA-MB-468 and MDA-MB-436 cell lines	Oncogene
Jin [132]	Breast	miR-10b	FOXO3a	48 tumor samples 48 normal samples MCF-7, MDA-MB-231, SK-BR-3, T47D and BT474 cell lines	Tumor suppressor
Xia [136]	Ovarian	miR-506-3p	FOXO3a	60 tumor samples 20 normal samples HO-8910PM, A2780, HO-8910, CAOV3, SKOV3, OVCA433, PEO1 and COC1cell lines	Tumor suppressor
Sang [137]	Breast	miR-182-5p	FOXO3a	230 tumor samples 44 normal samples MCF7 and T47D cell lines	Tumor suppressor
Zhu [138]	Ovarian	miR-148a	FOXO3	20 tumor samples 20 normal samples OVCAR3 and SKOV3 cell lines	Tumor suppressor
Li [139]	Cervical	miR-150	FOXO4	118 tumor samples 23 normal samples C-33A cell line	Oncogene
Hu [145]	Ovarian	miR-29c-3p	FOXP1	SKOV3 and A2780 cell lines	Tumor suppressor
Li [146]	Ovarian	miR-374b-5p	FOXP1	84 tumor samples 84 normal samples OVCAR3, 3AO, A2780 and SKOV3 cell lines	Tumor suppressor
Cheng [147]	Cervical	miR-449b-5p	FOXP1	84 tumor samples 84 normal samples HeLa, SiHa, ME180, CaSki, and C33A cell lines	Tumor suppressor
Qin [148]	Breast	miR-214-3p	FOXP2	10 tumor samples 10 normal samples MDA-MB-231, HCC1559, BT549, UACC-812, and MDA-MB-453 cell lines	Oncogene
Zhang [149]	Cervical	miR-185-3p	FOXP3	39 tumor samples 39 normal samples SiHa, HeLa, CaSki, HCC94 and C33A cell lines	Oncogene
Zhang [150]	Ovarian	miR-150-5p/3p	FOXP3	SKOV3, ES2, and A2780 cell lines	Tumor suppressor
Liu [158]	Breast	miR-146	FOXP3	20 tumor samples 20 normal samples MCF7, T47D, BT474, MDA-MB-468, and MDA-MB23 cell lines	Tumor suppressor
Wang [160]	Breast	miR-4316	FOXP4	41 tumor samples 21 normal samples BT474, MCF-7, MDA-MB-231, T47D and MDA-MB-453 cell lines	Tumor suppressor
Yang [161]	Breast	miR-296-5p	FOXP4	70 tumor samples 70 normal samples T47D, MCF-7, MDA-MB-231 and BT549 cell lines	Tumor suppressor
Han [166]	Breast	miR-937	FOXQ1	47 tumor samples 47 normal samples MDA-MB-231, MCF-7, BT-474 and SKBR3 cell lines	Tumor suppressor
Deng [167]	Endometrial	miR-202	FOXR2	90 tumor samples 40 normal samples KLE and AN3CA cell lines	Tumor suppressor
Zhang [168]	Ovarian	miR-1252	FOXR2	36 tumor samples 36 normal samples SKOV3 and HeyA-8 cell lines	Tumor suppressor

## FOXA, C, D, F, and G

**Fig. 1:**
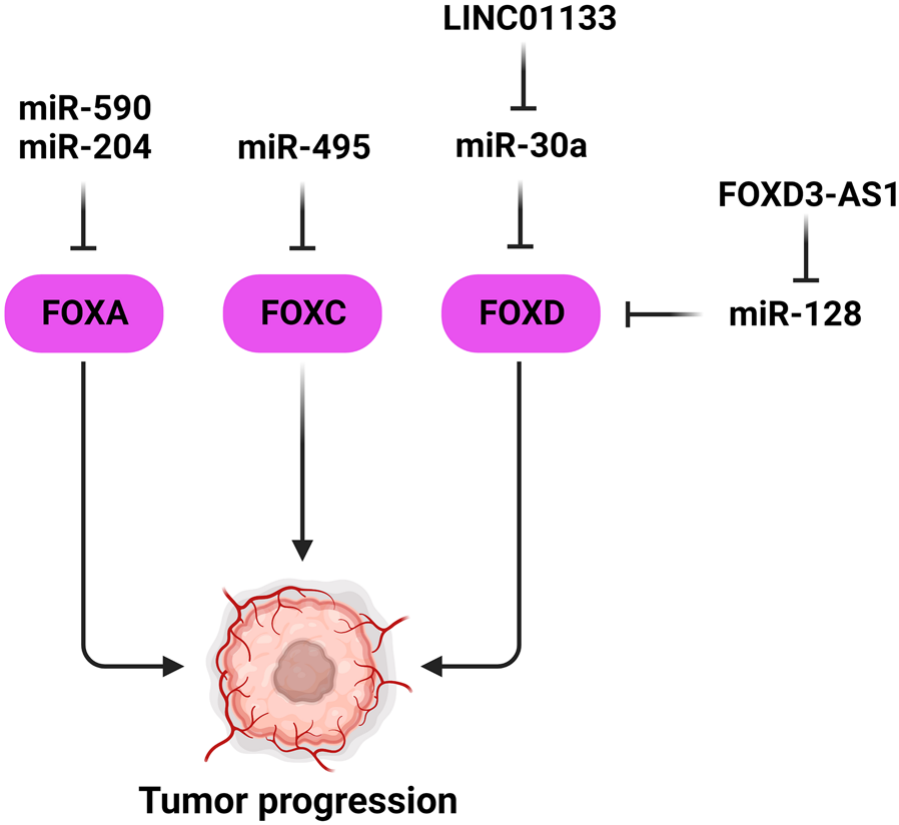
MicroRNAs have important roles in gynecological and breast tumors progressions by the FOXA, FOXC, and FOXD regulations. (Created with BioRender.com)

FOXA protein family plays pivotal roles in the endoderm and endoderm development [53]. They are expressed in various tissues including the mammary gland, pancreas, liver, and the prostate to regulate cellular differentiation and organ function [54]. FOXA, FOXC, and FOXD have key roles in progression of gynecological and breast tumor cells that can be regulation by miRNAs (Fig. 1). Down-regulation of miR-204 expression was correlated with metastasis and tumor stage in BC. MiR-204 suppressed BC cell proliferation and invasion, while promoted apoptosis by FOXA1 targeting [55]. There was miR-590-3p up-regulation in EOC tumor tissues and plasma samples that was significantly correlated with high-grade tumors. FOXA2 down-regulation and VCAN up-regulation were significantly correlated with reduced survival rates in EOC patients. MiR-590-3p significantly promoted EOC cell proliferation, invasion, and in vivo growth via FOXA2 targeting and VCAN up-regulation [56].

FOXC is involved in promotion of tumor angiogenesis, EMT, and invasion. FOXC2 induces HGF–MET signaling to promote colorectal tumor cell invasion [57]. It is also involved in regulation of tumor glycolysis and lipid metabolism [58, 59]. FOXC proteins are required for the cardiovascular system and kidney development [60]. FOXC1 and FOXC2 deletion has been correlated with abnormal lymphatic remodeling [61, 62]. There were FOXC1 up-regulation and miR-495 down-regulation in endometrial tumor tissues compared with healthy tissue. MiR-495 inhibited cell proliferation and migration in endometrial cancer through apoptosis induction. FOXC1 inhibited the endometrial tumor cell proliferation and migration. There was a negative correlation among miR-495 and FOXC1 and inhibition of endometrial tumor progression [63].

FOXD is a crucial factor during the kidney and neuronal development [64–66]. FOXD3 has tumor-suppressive functions and inhibits angiogenesis in neuroblastoma and non-small cell lung cancer; however, its deficiency triggers EMT and promotes aggressiveness in breast tumor cells [67–69]. Long non-coding RNAs (lncRNAs) are involved in cell proliferation, apoptosis, and invasion [70, 71]. There was a significant LINC01133 up-regulation in CC tissues compared with paired adjacent normal tissues that was correlated with the increase of T stage and negative HPV infection. LINC01133 enhanced CC cell migration and proliferation by the regulation of miR-30a-5p/FOXD1 axis [72]. LIM domain kinase 1 (LIMK1) plays a key role in cytoskeletal remodeling by stimulation of ROCK1, Rac/p21 activated kinase 1, and CDC42/MRCK signaling pathways [73, 74]. FOXD3-AS1 was significantly up-regulated in CC cells and tissues compared with normal cervical epithelial cell lines and margins, respectively. It was significantly correlated with poor differentiation and lymph node involvement in CC patients. FOXD3-AS1 down-regulation significantly suppressed CC tumorigenic behavior in comparison with the control group. FOXD3-AS1 enhanced the cancerous phenotype of CC cells by miR-128-3p sponging and LIMK1 up-regulation [75].

Forkhead box F2 (FoxF2) is involved in promotion of organ development, extracellular matrix (ECM) synthesis, and EMT. There was significant miR-182 up-regulation in triple negative breast cancer (TNBC) tissues and cells. It increased TNBC cells proliferation and metastasis by CDH1 and FOXF2 targeting [76]. ADAMTS9-AS2 down-regulation was correlated with poor survival rate, advanced FIGO stage, and lymph-node involvement in ovarian cancer (OC) patients. ADAMTS9-AS2 significantly reduced OC cell proliferation and EMT process by miR-182-5p sponging and FOXF2 up-regulation [77].

Transforming growth factor-β (TGF-β) is an important regulator of different biological processes, including self-renewal, tissues homeostasis, and tumor metastasis [78, 79]. It has a dual function as a tumor suppressor in normal cells and early carcinomas, while oncogene in advanced invasive tumor cells [80, 81]. Forkhead Box G1 (FOXG1) plays an important role in cortical development [82, 83]. It acts as an oncogene by suppressing TGF-b-mediated anti-proliferative responses in tumor cancer cells by p21WAF1/CIP1 down-regulation [84, 85]. MiR-200b was up-regulated in cervical tumor tissues compared with normal margins that was associated with tumor progression through FoxG1 targeting [86].

## FOXK, M, and N

**Fig. 2:**
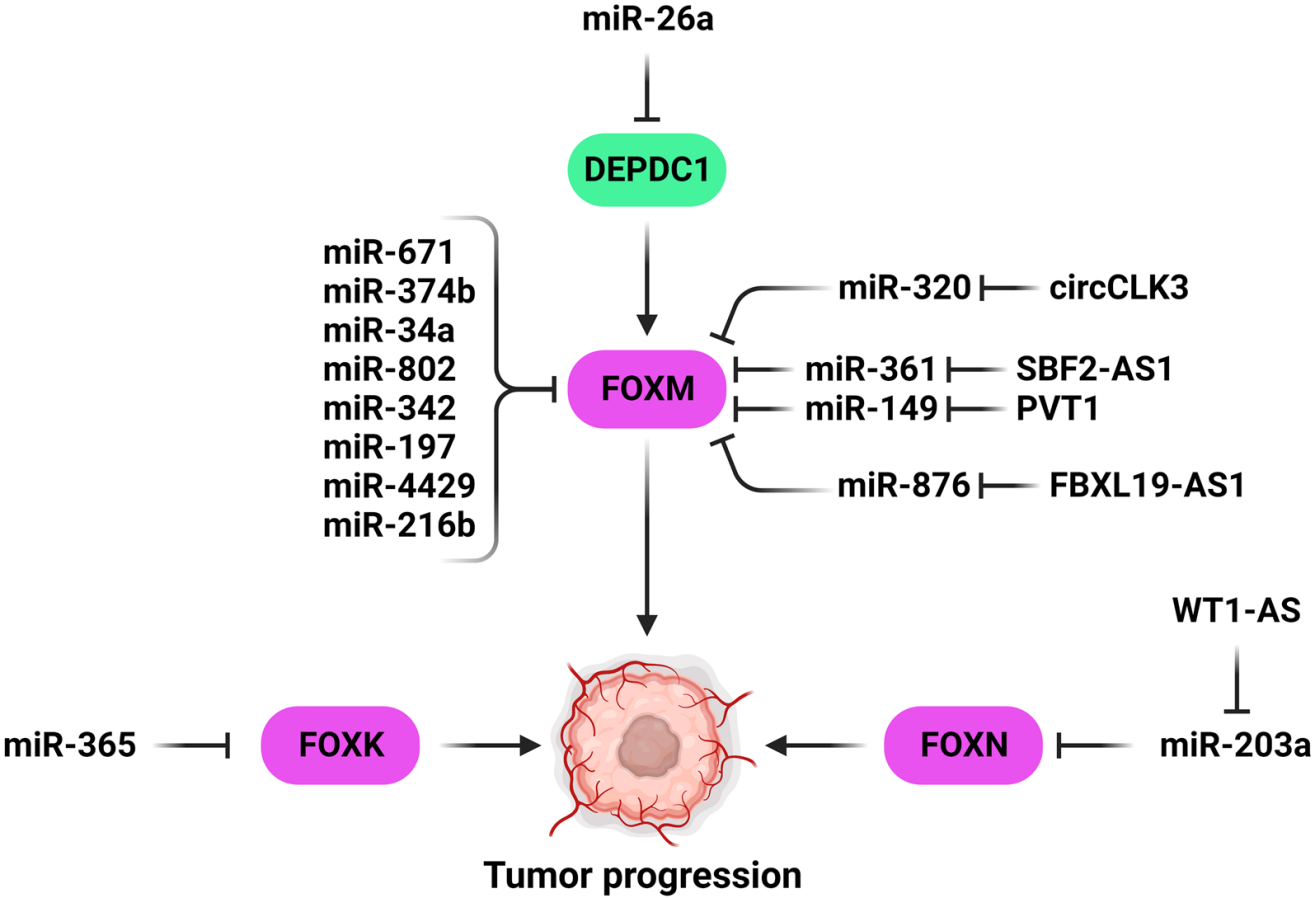
MicroRNAs have important roles in gynecological and breast tumors progressions by the FOXM, FOXK, and FOXN regulations. (Created with BioRender.com)

Forkhead Box Class K (FOXK) proteins subfamily mediate cell proliferation, differentiation, apoptosis, and DNA repair [87]. There was FOXK1 up-regulation in BC tissues and cell lines that was correlated with TNM stage, tumor size, and invasion. FOXK1 induced cell migration by EMT regulation in breast tumor cells. FOXK1 significantly increased breast tumor cell proliferation via facilitating G1/S transition. MiR-365-3p is the negative regulator of FOXK1 during BC progression [88]. FOXM, FOXK, and FOXN have key roles in cell cycle progression in gynecological and breast tumor cells that can be regulation by miRNAs (Fig. 2). FOXM1 is a key regulator of G2/M-specific proteins in different tumor types [89–91]. Triple-negative breast cancer is a kind of Basal-like BC without the expression of estrogen receptor (ER), HER2, and progesterone receptor (PR) [92]. TNBC has several clinical manifestations including higher invasiveness, larger tumor size, and tumor load and higher susceptibility to metastasis in comparison with other subgroups [93]. It is more common in young women that accounts 9–16% of cases [94]. FOXM1 is involved in regulation of DNA replication and cell cycle phase transition during normal cell proliferation and tumorigenesis [95–97]. FOXM1 is also involved in positive regulation of different transcription factors, including cyclin A, cyclin B, and polo-like kinase1. FOXM1 can also reduce nuclear accumulation of p21cip1 and p27kip1 as the CDK inhibitor proteins through their deterioration [98, 99]. DEP domain containing 1 (DEPDC1) is a transcriptional suppressor that promotes anti-apoptotic pathway by activating the NF-κB pathway [100]. DEPDC1 up-regulation was observed in TNBC tissue and enhanced cell proliferation. MiR-26b functioned as a negative regulator of DEPDC1 in TNBC cells and also FOXM1 enhanced stimulating effects of DEPDC1 on tumor growth. DEPDC1 increased TNBC cell proliferation through FOXM1 up-regulation [101]. Eukaryotic elongation factor 2 kinase (eEF2K) negatively mediates phosphorylation and inactivation of eEF2, the protein that facilitates the elongation step of protein synthesis [102, 103]. eEF2K acts as a negative regulator of cell growth, protein synthesis, and translation. It is highly expressed in different cancers, and also is activated under stress conditions, including energy depletion or nutrient starvation [104]. MiR-34a down-regulation was correlated with longer overall survival of TNBC patients. MiR-34a was negatively associated with the expression of eEF2K which was also correlated with shorter survival of patient. MiR-34a inhibited cell growth and invasion via FOXM1/eEF2K axis in TNBC cells [105]. FOXM1 has a key role in regulation of angiogenesis and EMT [106]. MiR-802 suppressed BC cell proliferation via FOXM1 targeting [107]. Down-regulation of miR-342-3p reduced cervical tumor cell proliferation, growth, and migration by targeting FOXM1 [108]. There was miR-4429 down-regulation in CC tissues that was contributed with poor prognosis. MiR-4429 suppressed the CC cell proliferation and EMT, while promoted apoptosis through FOXM1 targeting [109]. PVT1 up-regulation was correlated with a shorter survival in OC patients. PVT1 suppressed OC cell apoptosis, while promoted cell viability and drug resistance via miR-149-5p/FOXM1 axis [110]. CircCLK3 promoted CC cell proliferation and invasion by miR-320a sponging and FoxM1 up-regulation. There was a significant circCLK3 up-regulation in CC tissues compared with normal samples. CircCLK3 was also significantly correlated with advanced FIGO stages and depth of stromal invasion [111]. There was significant SBF2-AS1 up-regulation in CC cell lines and tissues that was associated with lymph node involvement and progressive FIGO stage. Down-regulation of SBF2-AS1 significantly decreased CC cells survival. SBF2-AS1 suppression led to cell cycle arrest and reduced in-vivo growth, while increased apoptosis in CC cells. SBF2-AS1 promoted the CC progression by miR-361-5p sponging and FOXM1 up-regulation [112]. FBXL19-AS1 was up-regulated in BC cancer cells. Down-regulation of FBXL19-AS1 reduced BC cell proliferation, while increased apoptosis. FBXL19-AS1 promoted BC progression via miR-876/FOXM1 axis [113]. MiR671-5p suppressed cell proliferation and invasiveness by FOXM1 targeting. It down-regulated the genes that were involved in cell proliferation, such as GINS2, CDK2, and MCM10. MiR-671-5p was involved in cell cycle regulation through FOXM1 targeting which suppressed CDK2 and CCNB1 [114]. An inverse association has been reported between miR-216b and FOXM1 expression in CC cells. MiR-216b suppressed the CC cell proliferation by pRb, c-Myc, and CCND1 down-regulations, which were downstream targets of FOXM1 [115]. MiR-197, miR-374b, and miR-320 were also considered as the tumor suppressors that inhibited the CC cell proliferation and motility via FOXM1 suppression [116–118]. WT1-AS was significantly down-regulated in CC tissues and cell lines. WT1-AS inhibited the CC cell growth and motility by miR-203a-5p sponging that resulted in FOXN2 up-regulation [119].

## FOXO

**Fig. 3:**
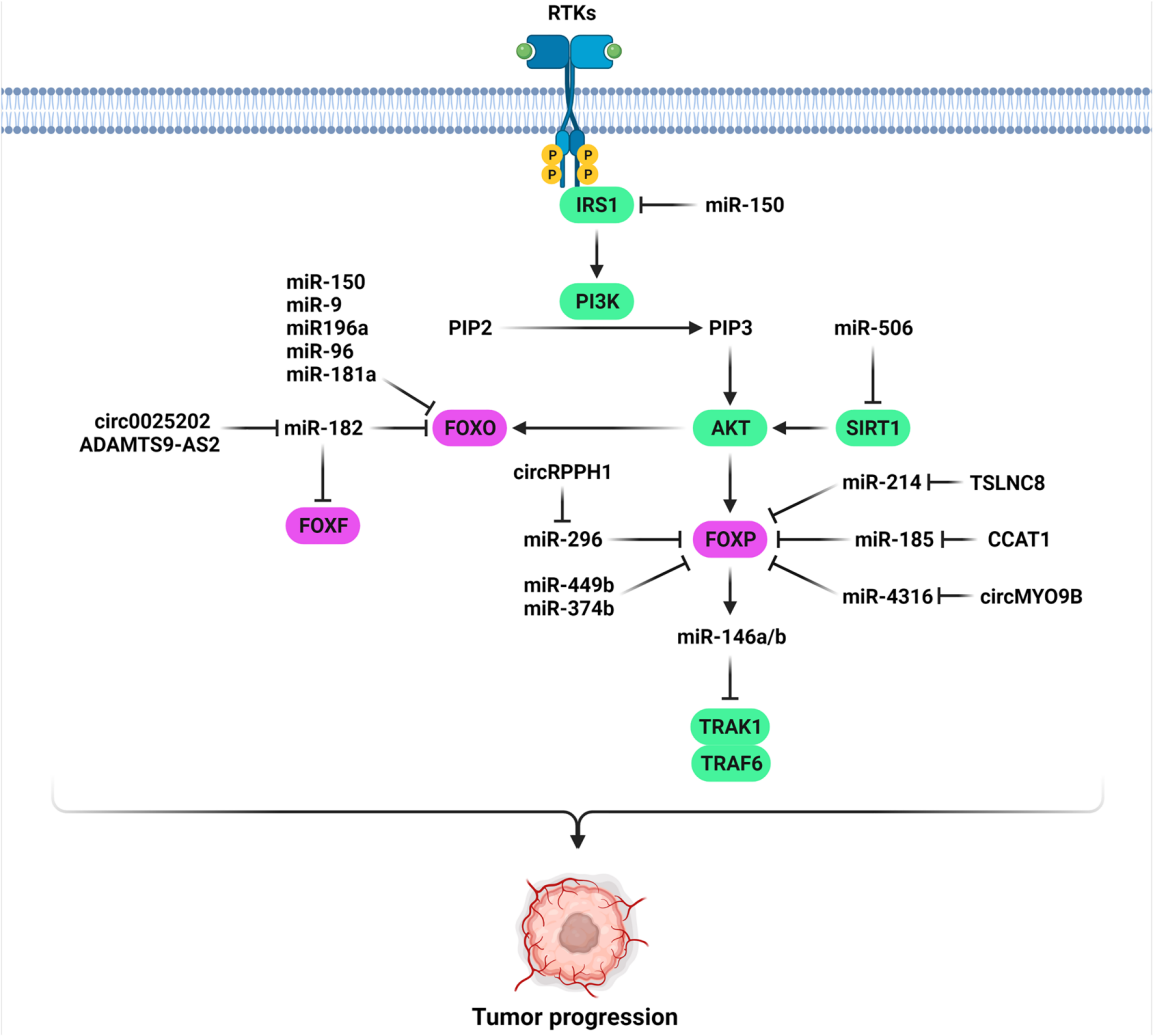
FOXO and FOXP are the main effectors of PI3K/AKT pathway that can be regulated by microRNAs during gynecological and breast tumor progressions. (Created with BioRender.com)

Forkhead box O (FOXO) protein family are the critical regulators of PI3K/Akt signaling which are involved in cell differentiation, cell cycle regulation, and tumor progression [120–122]. FOXO1 acts as a crucial downstream effector in PI3K/Akt signaling pathway. Activation of Akt, leads to phosphorylation of FOXO1 and its localization in the cytoplasm instead of the nucleus, consequently inhibition of FOXO1-regulated genes. FOXO1 target genes are involved in different biological processes, including carcinogenesis and cell cycle modulation (Fig. 3). It has been demonstrated that miR-181a was significantly up-regulated in CC cells in comparison with healthy cervical epithelium cell, and miR-181a played a key regulatory role in growth and invasion of CC cells via the PTEN/AKT/FOXO1 pathway. Inhibition of miR-181a enhanced p21 and p27 expression. Down-regulation of miR-181a significantly inhibited CC cell invasion by enhancing TIMP3 expression and down-regulation of MMP6 expression [123]. CCND1 is a key regulator of cell cycle progression that is also known as a growth-promoting factor in the G1 phase [124]. There was miR-96 up-regulation in CC tissues that was significantly associated with tumor staging, lymph nodes involvement, and differentiation. MiR-96 enhanced G1/S-phase transition, cell proliferation, and colony formation by FOXO1 targeting. Suppression of miR-96 promoted apoptosis and suppressed cell proliferation by up-regulating the p21 and p27 in CC cells [125]. Suppression of miR-135b reduced CC cell growth through FOXO1, p21, and p27 up-regulations while CCND1 down-regulation [124]. There was significant miR-196a up-regulation in CC tissues that was contributed with prognosis and stage. MiR-196a enhanced CC cell proliferation by p21Kip1 and FOXO1 targeting [51]. DNA methyl transferase 3 beta (DNMT3B) is a key factor of epigenetic regulation during embryogenesis and imprinting that is also upregulated in different tumors [126, 127]. MiR-29c down-regulation was reported in BC in comparison with normal tissues. MiR-29c suppressed tumor growth and migration by DNMT3B targeting. DNMT3B was necessary for the methylation and down-regulation of TIMP3, which enhanced BC progression through the TIMP3/STAT1/FOXO1 axis [128]. MiR-9 increased BC cell proliferation and migration through FOXO1 targeting and CDH1 down-regulation [129].

Twist-related protein 1 (TWIST-1) is a transcription factor involved in EMT induction [130, 131]. FOXO3a suppressed EMT and metastasis through regulation of miR-10b and CADM2 expression and TWIST-1 down-regulation in BC cells [132]. Sirtuin 1 (SIRT1) belongs to the class-III histone deacetylase (HDAC) participated in different physiological and pathological processes, such as gene regulation, DNA repair, cell proliferation, aging, and tumorigenesis. It has also critical roles in the epigenetic modulation of tissue homeostasis and various diseases through histone and non-histone deacetylation [133]. It has oncogenic or tumor suppressor functions in different cancers [134, 135]. MiR-506-3p inhibited OC cell proliferation, while induced apoptosis through SIRT1 suppression. FOXO3a and AKT were also the downstream targets of SIRT1. Therefore, miR-506-3p reduced the expression levels of p-AKT and p-FOXO3a in OC cells. Moreover, SIRT1 up-regulation conversed the suppression ability of miR-506-3p on p-AKT and p-FOXO3a expression [136]. It has been demonstrated that miR-940 was markedly up-regulated in BC tissues and cells which was associated with decreased survival in BC patients. MiR-940 enhanced cell invasiveness and proliferation in BC via regulation of FOXO3 [125]. Circ-0025202 promoted cell apoptosis and TAM sensitivity and suppressed BC cell colony formation and proliferation. Down-regulation of circ-0025202 was associated with histological grade and metastasis to lymph nodes, proposing that it functioned as a significant regulator and tumor suppressor in HR-positive breast cancer. Circ-0025202 was involved in tumor progression and regulation of TAM sensitivity through miR-182-5p/FOXO3a axis [137]. MiR-148a inhibited ovarian tumor cell viability and invasion, while induced apoptosis by FOXO3 targeting [138]. A significant miR-96 up-regulation was shown in BC tissues in comparison with normal samples. MiR-96 increased BC cell proliferation by FOXO3a targeting that down-regulated the p27Kip1 and p21Cip1, while up-regulated CCND1 [52]. MiR-150 was significantly over expressed in CC patients compared to normal tissues that were correlated with the processed stages of cancer. MiR-150 enhanced CC cell growth and survival in via FOXO4, BIM, and FASL targeting. It also promoted the cell cycle progression from the G1/G0 to S phase in CC cells by p27 down-regulation while CCND1 up-regulation [139].

## FOXP, Q, and R

FOXP subfamily is involved in cancer progression and embryonic development through interacting with noncoding RNAs and signaling pathways [83, 140]. They are one of the main effectors in PI3K/AKT pathway (Fig. 3). FOXP1/2/4 are expressed in brain, while FOXP3 is mainly expressed in T regulatory cells. FOXP family functions as oncogene or tumor suppressor in different types of cancer [21, 141, 142]. LC3 is known as a homologue of yeast ATG8 in mammalian cells [143]. LC3-II ratio and LC-II to LC3-I amount display the quantity and content of autophagy [144]. It was indicated that the elevated LC3-II/LC3-I ration up-regulated the Beclin1 and MDR-1, while down-regulated the p62 in CDDP-resistant cells, indicating that the DDP resistance was correlated with autophagy in ovarian cancer. MiR-29c-3p reduced autophagy and CDDP resistance via FOXP1/ATG14 targeting in OC cells [145].

MiR-374b-5p down-regulation was correlated with poor prognosis in ovarian tumor tissues. MiR-374b-5p played as a tumor suppressor by regulation of ovarian tumor cell proliferation, EMT, and CDDP sensitivity via FOXP1 targeting [146]. A direct association was shown between the miR-449b-5p expression level and overall survival rate of CC patients. MiR-449b-5p suppressed the CC cell proliferation and invasion via FOXP1 targeting [147]. There was TSLNC8 down-regulation in BC cell lines and tissues. TSLNC8 significantly suppressed tumor growth and G1/S phase transition in BC cells by miR-214-3p sponging and FOXP2 up-regulation [148].

SOX2 and CCAT1 up-regulations were observed in CC tissues and cells which were correlated with LNM, tumor size, and advanced FIGO. SOX2 and CCAT1 silencing reduced CC stem cell proliferation and invasion, while promoted apoptosis. CCAT1 inhibited the CC stem cell proliferation and self-renewal by miR-185-3p sponging and FOXP3 up-regulation [149]. There was miR-150-5p/3p down-regulation in OC in comparison with normal tissues. MiR-150 significantly reduced OC cell proliferation and invasion, while increased apoptosis through IGFIR and IRS1 targeting. FoxP3-miR-150 axis inhibited the OC progression through IGF1R/IRS1 feedback loop in which PI3K/AKT pathway reduced the levels of FoxP3 expressions [150]. Tumor necrosis factor receptor-related factors (TRAFs) are a class of cytoplasmic adaptor proteins that link tumor necrosis factors to the Toll-like/IL-1 receptor (TLR/ILR) superfamily [151]. TRAF6 overexpression has been observed in different tumor types which can promote tumor progression by regulating various signaling pathways involved in cell proliferation and invasion [152]. Interleukin-1 receptor-associated kinase (IRAK) is a serine/threonine kinase involved in regulation of the IL-1R signaling pathway. It is also a key effector of the TLR signaling pathway [153, 154]. IRAK1 is participated in the formation and development of different myeloid malignancies or tumors [155–157]. It has been revealed that up-regulation of miR-146a/b by FOXP3 led to inhibition of IRAK1 and TRAF6 that resulted in suppression of NF-κB and consequently tumor growth inhibition in BC. FOXP3 targeted miR-146a via two forkhead-binding motifs which were located in proximal site of the miR-146a promoter. Tumor suppressor function of FOXP3 was partially inhibited by miR-146a/b negative regulators [158]. Circular RNAs (circRNAs) are a type of noncoding RNAs, defined as continuous loops that are closed covalently and derive from mRNA splicing [159]. There was circMYO9B up-regulation in BC tissues. Knockdown of circMYO9B inhibited BC cell progression, invasion, and migration by miR-4316 sponging and FOXP4 up-regulation [160]. CircRPPH1 was significantly up-regulated in BC tissues and cells, which was associated with lymph node involvement and tumor stage. CircRPPH1 promoted BC progression through miR-296-5p sponging and FOXP4 up-regulation. Down-regulation of circRPPH1 inhibited cell proliferation, metastasis, and glycolysis in BC cells [161].

Forkhead box Q1 (FOXQ1) is involved in gastric epithelial differentiation [162]. FOXQ1 has oncogenic role in different types of cancer [163]. FOXQ1 induces tumor angiogenesis, cell proliferation, resistance to chemotherapy, and EMT [163–165]. There was miR-937 down-regulation in BC cell lines and tissues that was associated with TNM stage and lymph node involvement. Down-regulation of miR-937 decreased overall survival in BC patients. MiR-937 inhibited tumor development through FOXQ1 targeting [166]. It has been reported that miR-202 was significantly down-regulated in endometrial adenocarcinoma (EAC) tissues in comparison with the normal samples. Down-regulation of miR-202 was linked to overall survival rate. MiR-202 significantly suppressed tumor growth through FOXR2 inhibition in EAC [167]. There was significant circ-CELSR1 up-regulation in PTX-resistant ovarian tumor tissues and cell lines. It increased OC progression by miR-1252 sponging and FOXR2 up-regulation. Suppression of circCELSR1 increased the PTX sensitivity of ovarian tumor cells [168].

## Conclusions

Gynecological and breast tumors are one of the leading causes of cancer-related mortality among women. Late diagnosis is one of the main reasons for treatment failure and high mortality in these patients. Therefore, the introduction of early diagnostic markers can be significantly effective in the management and control of patients in the early stages. FOX protein family has critical role in development and progression of these tumors. On the other hand, miRNAs as non-invasive factors play an important role in regulation of FOX function. Therefore, in the present review, we assessed the role of miR/FOX axis during the progression of these tumors. It has been reported that miR/FOX axis has mainly a tumor suppressor role in these tumors. MicroRNAs were mainly involved in progression of these tumors through FOXM, FOXP, and FOXO. The present review paves the way to suggest a non-invasive diagnostic panel marker based on miR/FOX axis in gynecological and breast cancers. However, further clinical studies on the circulating levels of miRNAs are required to introduce them as the efficient non-invasive tumor markers. Although, miR/FOX axis can be also suggested as a therapeutic target in gynecological and breast cancer patients, further animal studies and clinical trials are required to bring miR/FOX axis into the clinics as an efficient therapeutic target in gynecological and breast cancer patients.

## Data Availability

Not applicable.

## Abbreviations

AGO: Argonaute
BC: Breast cancer
CC: Cervical cancer
circRNAs: Circular RNAs
DNMT3B: DNA methyl transferase 3 beta
EAC: Endometrial adenocarcinoma
ER: Estrogen receptor
eEF2K: Eukaryotic elongation factor 2 kinase
XPO5: Exportin 5
ECM: Extracellular matrix
FOX: Fokhead box
FOXK: Forkhead box class K
FoxF2: Forkhead box F2
FOXO: Forkhead box O
FOXQ1: Forkhead box Q1
FHD: Forkhead domain
HDAC: Histone deacetylase
IRAK: Interleukin-1 receptor-associated kinase
LIMK1: LIM domain kinase 1
lncRNAs: Long non-coding RNAs
miRNAs: MicroRNAs
OC: Ovarian cancer
pre-miRNAs: Precursor miRNAs
pri-miRNAs: Primary miRNAs
PR: Progesterone receptor
RISC: RNA-induced silencing complex
SIRT1: Sirtuin 1
TGF-β: Transforming growth factor-β
TNBC: Triple negative breast cancer
TRAFs: Tumor necrosis factor receptor-related factors
TWIST-1: Twist-related protein 1
UTR: Untranslated regions
